# Impact of corpus callosum fiber tract crossing on polarimetric images of human brain histological sections: *ex vivo* studies in transmission configuration

**DOI:** 10.1117/1.JBO.28.10.102908

**Published:** 2023-09-12

**Authors:** Deyan Ivanov, Lu Si, Leonard Felger, Theoni Maragkou, Philippe Schucht, Marie-Claire Schanne-Klein, Hui Ma, Razvigor Ossikovski, Tatiana Novikova

**Affiliations:** a Institut Polytechnique de Paris, École Polytechnique, CNRS, LPICM, Palaiseau, France; b Tsinghua University, Tsinghua-Berkeley Shenzhen Institute, Shenzhen, China; c Bern University Hospital, University of Bern, Inselspital, Department of Neurosurgery, Bern, Switzerland; d University of Bern, Institute of Tissue Medicine and Pathology, Bern, Switzerland; e Institut Polytechnique de Paris, École Polytechnique, Inserm, CNRS, LOB, Palaiseau, France; f Tsinghua University, Department of Physics, Beijing, China; g Florida International University, Department of Biomedical Engineering, Miami, Florida, United States

**Keywords:** corpus callosum, brain fiber crossing, Mueller polarimetry, decomposition algorithms, image processing

## Abstract

**Significance:**

Imaging Mueller polarimetry is capable to trace in-plane orientation of brain fiber tracts by detecting the optical anisotropy of white matter of healthy brain. Brain tumor cells grow chaotically and destroy this anisotropy. Hence, the drop in scalar retardance values and randomization of the azimuth of the optical axis could serve as the optical marker for brain tumor zone delineation.

**Aim:**

The presence of underlying crossing fibers can also affect the values of scalar retardance and the azimuth of the optical axis. We studied and analyzed the impact of fiber crossing on the polarimetric images of thin histological sections of brain corpus callosum.

**Approach:**

We used the transmission Mueller microscope for imaging of two-layered stacks of thin sections of corpus callosum tissue to mimic the overlapping brain fiber tracts with different fiber orientations. The decomposition of the measured Mueller matrices was performed with differential and Lu–Chipman algorithms and completed by the statistical analysis of the maps of scalar retardance, azimuth of the optical axis, and depolarization.

**Results:**

Our results indicate the sensitivity of Mueller polarimetry to different spatial arrangement of brain fiber tracts as seen in the maps of scalar retardance and azimuth of optical axis of two-layered stacks of corpus callosum sections The depolarization varies slightly (<15%) with the orientation of the optical axes in both corpus callosum stripes, but its value increases by 2.5 to 3 times with the stack thickness.

**Conclusions:**

The crossing brain fiber tracts measured in transmission induce the drop in values of scalar retardance and randomization of the azimuth of the optical axis at optical path length of 15  μm. It suggests that the presence of nerve fibers crossing within the depth of few microns will be also detected in polarimetric maps of brain white matter measured in reflection configuration.

## 1 Introduction

Recent scientific advances of polarimetry have expanded its applications from material characterization and metrology,^1^^,^^2^ to biomedical diagnosis,^3^^–^^10^ and made it possible to combine polarimetry with the artificial intelligence algorithms.^11^^–^^19^ However, it is challenging to interpret directly the meaning of the elements of experimental Mueller matrix of biological tissue, especially without the prior knowledge of a physical model accurately describing the measured sample. Hence, the important task is the choice of the most suitable Mueller matrix decomposition algorithm, which needs to take into account the experimental geometry, type of measured sample, and the initial assumptions on whether the polarimetric effects occur continuously or sequentially throughout the sample. It is also challenging for both physicists and physicians to combine efforts to adopt unambiguously an optical technique and instrumentation for *in vivo* practical use,^20^^,^^21^ especially for early and accurate diagnosis, when there is a suspicion of malignancy. If such an optical technique can be non-invasively employed with minimum false positive or false negative results and medical doctors can utilize it at will in their medical practice, the diagnostic accuracy could be improved, followed by an increased life expectancy and quality.

A typical example is the case of brain surgery, where the precise localization of a tumor zone border is needed for the most complete and safe resection of brain tumor. In case a residual brain tissue with malignancy is left without treatment, tumor recurrence and the survival of a patient will be at stake.^22^^–^^24^ On the other hand, if a large part of adjacent tissue is removed, it may affect brain functions, leading to unwanted complications.^25^ In clinical practice, the conventional methods to localize brain tumors during neurosurgery include intra-operative magnetic resonance imaging^26^ and 5-aminolevulinic acid (5-ALA) intra-operative fluorescence imaging assisted surgery.^27^ However, both imaging techniques have limitations in acquisition time and detection of the border of low grade tumors, respectively. As white matter of healthy brain of mammals comprises nerve fiber bundles, optical and structural anisotropy are present.^28^^,^^29^ The brain tumor cells grow chaotically and break the anisotropy of healthy brain white matter. Our prior studies showed the potential of multispectral wide-field imaging Mueller polarimetry operating in reflection to detect the optical anisotropy by visualizing the in-plane orientation of the nerve fiber bundles^7^^,^^30^^–^^33^ by applying Lu–Chipman decomposition of Mueller matrix images^34^ and to explore the absence of such anisotropy as an optical marker for brain tumor. An open question to address is the capability of imaging Mueller polarimetry to detect the underlying crossing nerve fibers, because their presence within the volume of the optically probed brain tissue should affect the polarimetric maps of retardance and may provide information for a neurosurgeon on how much tissue can still be removed without damaging the sensitive zones of a brain.

In this study, we explore the impact of brain fiber crossing on the polarimetric maps of scalar retardance, azimuth of the optical axis, and depolarization. We measured the stacks of differently oriented thin histological sections of human brain corpus callosum with the transmission Mueller microscope, because the corpus callosum serves as a connection between the two brain hemispheres and has a well-defined orientation of fiber bundles.^35^ Both differential^36^ and Lu–Chipman^34^ decomposition algorithms were used for Mueller matrix data post-processing to obtain the polarimetric maps and to compare the performance and applicability of both decompositions. The results confirm the capability of imaging Mueller polarimetry to detect the presence of the underlying crossing fibers at the depth of few microns.

## 2 Materials and Methods

### 2.1 Brain Corpus Callosum Sections

**Fig. 1:**
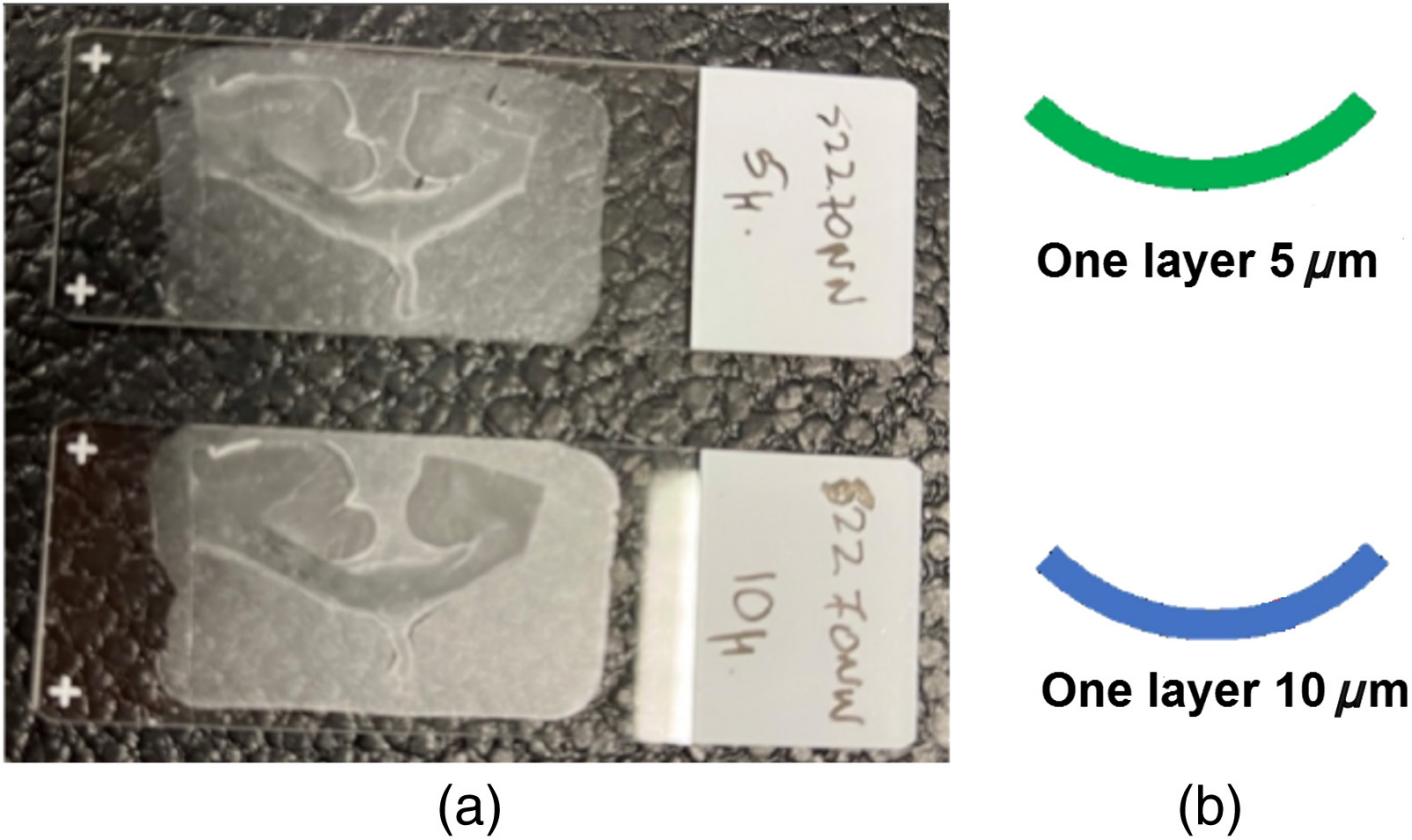
Thin histological sections of corpus callosum: (a) photo of the glass slides with the coronal brain tissue sections of 5  μm (top) and 10  μm (bottom) nominal thicknesses and (b) schematic layout of both corpus callosum sections.

We used a formalin-fixed human brain obtained from the autopsy of an anonymous donor. A waiver for ethical approval was obtained from the Ethics Committee of the Canton of Bern (BASEC-Nr: Req-2021-01173). We excised a 2.5×1.5×0.5  cm coronal section of the corpus callosum in the central area. Subsequently, the formalin-fixed specimen was embedded in paraffin. This block was then cut in sections of different thicknesses (e.g., 5  μm and 10  μm) for polarimetric measurements. The photo and schematic layout of the standard microscopy glass slides with thin (5  μm and 10  μm) histological sections of corpus callosum embedded in paraffin is shown in Fig. 1.

**Fig. 2:**
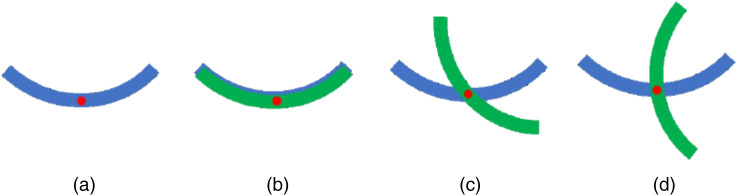
Schematics of the spatial arrangement of the histological sections with red spot showing the location of the measurement site: (a) single layer of 10  μm nominal thickness, (b) two parallel overlapped layers of 10  μm and 5  μm of nominal thickness, (c) top layer was rotated by ∼45 deg, and (d) top layer was rotated by ∼90  deg.

The schematics of the spatial arrangement of corpus callosum thin sections during the measurements with transmission Mueller microscope (see Sec. 2.2) is presented in Fig. 2. First, we measured the single layer of 10  μm nominal thickness, then the stack of two superimposed layers aligned parallel [Fig. 2(b)], then crossed at 45 deg [Fig. 2(c)] and finally at 90 deg [Fig. 2(d)].

The presence of glass slides did not affect our measurements, because the measured Mueller matrix of glass without tissue was close to that of air (i.e., the identity matrix), with the errors in normalized Mueller matrix elements <1%.

### 2.2 Mueller Microscope

**Fig. 3:**
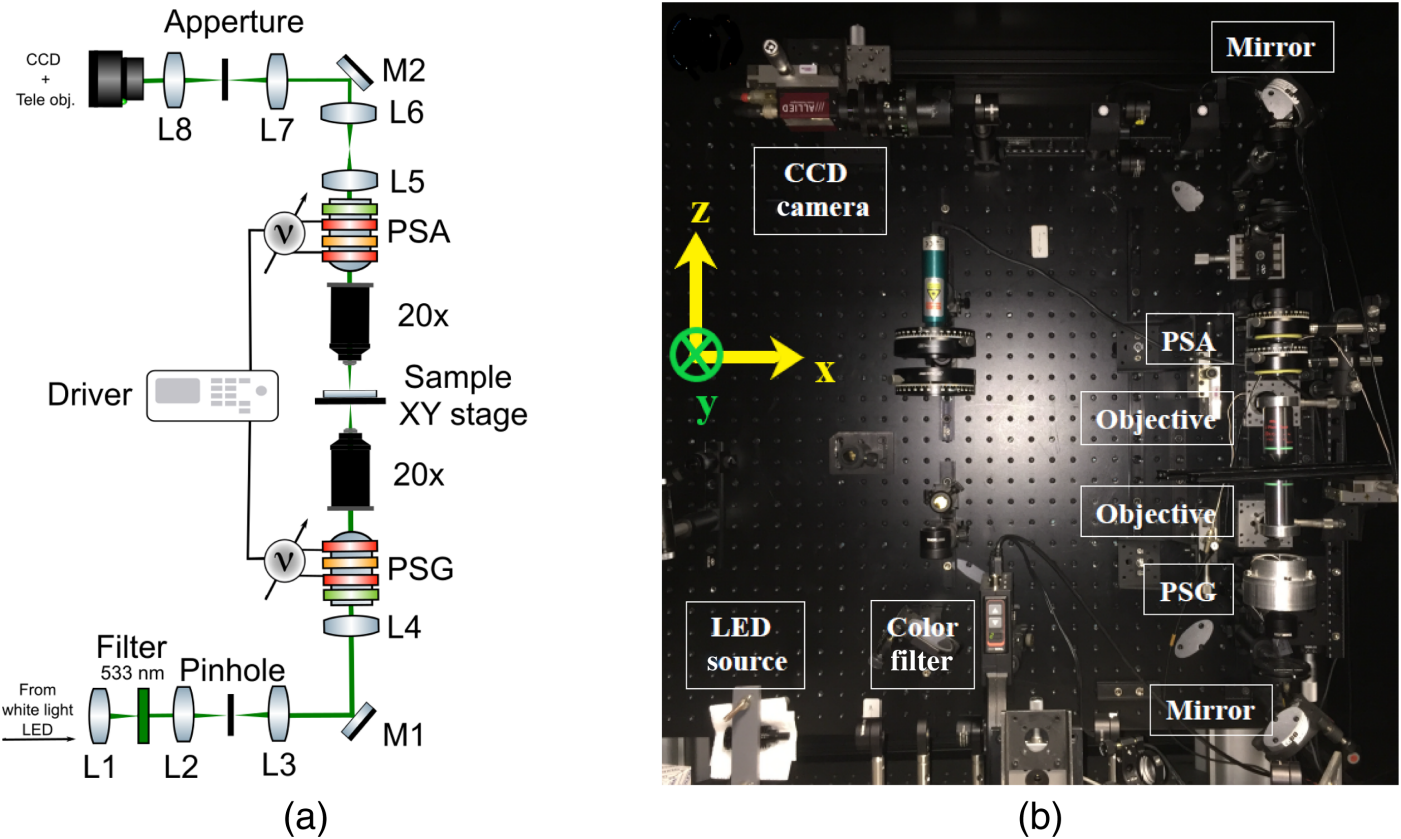
Experimental Mueller microscopy set-up in transmission geometry: (a) optical layout: achromatic lenses L1 to L8 (f=30, 50, 60, 100, 100, 75, 80, and 75, respectively); aluminium mirrors M1 and M2; PSG, polarization state generator; and PSA, polarization state analyser. (b) Side view of the system.

A custom-built Mueller microscope^37^ was used to measure the complete Mueller matrices of all brain samples in transmission geometry. The layout of the instrument and its photo in side view are shown in Fig. 3.

White-light 2.8 W light-emitting diode (LED) was chosen as a light source followed by a band-pass color filter (wavelength 550 nm and full width at half maximum (FWHM) 20 nm) to select the wavelength of a probing light beam. A 500-μm spatial filter (pinhole) was inserted in the illumination arm to control simultaneously the size and the angular aperture of a light beam. By aligning the pinhole to the optical axis of the first objective (Nikon, 20× magnification, NA 0.45), a sample can be illuminated at normal incidence. In accordance to the Koehler configuration, L3 serves as a condenser lens to illuminate the sample with uniform intensity.

Both the polarization state generator (PSG) and the polarization state analyzer (PSA) are comprised of the identical optical elements, but arranged in a reverse order. In short, they include a linear polarizer and two ferroelectric liquid crystal (FLC) retarders (Meadowlark FPR-200-1550) with a quarter-wave retarder placed between them. By varying the voltage applied to the FLC retarders, the orientation of their fast optical axis is switched between 0 deg and 45 deg. This approach for polarization modulation assures the generation of all optimal polarization states required to obtain the complete Mueller matrix of a specimen under study. The light scattered from a sample is collected by another objective (Nikon, 20× magnification, NA 0.45) and with the help of L4 lens the microscopic image can be formed in the real plane. The condenser and the imaging optics have always been kept identical to each other to match their respective numerical apertures. On the other hand, the passing of the light beam through the retractable Bertrand lens L6 allows to switch between the real and the Fourier imaging planes if needed. The latter configuration was used during the calibration stage for achieving more precise optical alignment. To monitor the image preview and to capture the images of interest, a telephoto lens was coupled and set to infinity to matrix photodetector (16-bit, single-channel, CCD camera AV Stingray F-080B). The Mueller microscope was calibrated using the eigenvalue calibration method, described in details elsewhere.^38^^,^^39^ For all measurements the field of view was set to ∼600  μm. Measuring the brain tissue histological sections with different thicknesses caused variations in the intensity detected by the sensor of the CCD camera. To avoid over- or under-exposure all measurements were conducted in the linear response range of the detector by varying the exposure time for each sample, while the gain was always kept at minimum. Before applying any of the decomposition algorithms to the recorded Mueller matrices the influence of the glass substrate was taken into account and corrected accordingly.

## 3 Mathematical Framework for the Mueller Matrix Data Decomposition

In this section, we describe explicitly the theory behind the decomposition algorithms used in this study. Let M denote an experimentally measured Mueller matrix, which has been filtered according to the Cloude’s physical realizability criterion.^40^^,^^41^ In that case, M can be used as an input for decomposition algorithm to extract the polarization signature of the sample under study, encoded in M. Depending on the initial assumptions for the turbid media, one may use different decomposition algorithms. In this work, two decomposition methods^34^^,^^36^ were used with the experimental data and they are described briefly in the following subsections.

### 3.1 Differential Decomposition

Assuming transversely homogeneous and longitudinally non-homogeneous turbid media (i.e tissue section placed on a glass slide with known thickness), one can use the following mathematical representation: dM=mMdz to describe the polarized light-tissue interactions. Each scattering event will be followed with polarization and/or depolarization along the direction of light propagation z. Then, according the the former differential equation, the initial Mueller matrix will be modified by factor of m. In this case, this is another 4×4 matrix, known as differential Mueller matrix, containing mean and mean squared values of polarization properties, likewise: $m=m_{m}+m_{u}=\langle m\rangle +\langle \Delta m^{2}\rangle z$ (the spatial averaging ⟨⟩ is performed within the transverse plane). Substituting m and solving the differential equation with respect to the boundary conditions, yields the following solution:^36^

$$\ln\;M=\langle m\rangle z+\frac{1}{2}\langle \Delta m^{2}\rangle z^{2}.$$ (1)

From here, the matrix logarithm could be computed by first solving the eigenvalue-eigenvector problem for M and then forming $D=\mathrm{diag}[\ln(d_{i})]$, where $d_{i}$ are the eigenvalues of M. Finally using the matrix V comprised of the eigenvectors of M, the matrix logarithm and the two counterparts of m can be calculated as

$$L\equiv \ln\;M=VDV^{-1},\;m_{m}=\frac{1}{2}(L-GL^{T}G),\;m_{u}=\frac{1}{2}(L+GL^{T}G),$$ (2)

where G=diag(1,−1,−1,−1) is the Minkowski metric tensor. For clarity, both the polarizing and the depolarizing tensors can be summarized as^37^

$$m_{m}=[\begin{array}{cccc} 0 & p_{1} & p_{2} & p_{3}\\ p_{1} & 0 & p_{6} & p_{5}\\ p_{2} & -p_{6} & 0 & p_{4}\\ p_{3} & -p_{5} & -p_{4} & 0 \end{array}],\;m_{u}=[\begin{array}{cccc} {\begin{array}{c} \alpha \end{array}}_{0} & d_{1} & d_{2} & d_{3}\\ -d_{1} & \alpha_{1} & d_{6} & d_{5}\\ -d_{2} & d_{6} & \alpha_{2} & d_{4}\\ -d_{3} & d_{5} & d_{4} & \alpha_{3} \end{array}],$$ (3)

where the notations used denote elementary polarization properties. They are the following: $p_{1}$ – linear dichroism (LD) along x-y axis, $p_{2}$ – LD along ±45  deg axis, $p_{3}$ – circular dichroism (CD), $p_{4}$ – linear birefringence (LB) along x-y axis, $p_{5}$ – LB along ±45  deg axis (${\mathrm{LB}}^{\prime }$), and $p_{6}$ – circular birefrigence. When the medium is depolarizing, all non-diagonal elements of $m_{m}$ are the mean values of the above-mentioned elementary properties. For the depolarizing tensor $m_{u}$, the non-diagonal elements $d_{i}$ are uncertainties of the same elementary properties. Additionally, on the main diagonal the values denote depolarizing factors $\alpha_{i}$ (provided that $\alpha_{0}=0$). Again, $\alpha_{1}$ is along the x-y axis, $\alpha_{2}$ is along the ±45° axis, and $\alpha_{3}$ is the circular component. Sometimes, $\alpha_{1}$, $\alpha_{2}$, and $\alpha_{3}$ are used as the components of anisotropic absorption and are denoted as LA, ${\mathrm{LA}}^{\prime }$, and CA, while $\alpha_{0}$ is the isotropic component.^36^ By virtue of Eq. (4), one can obtain the net scalar retardance (rotationally invariant) – $R_{t}$, the total depolarization – $\alpha_{t}$ (provided that $\alpha_{0}=0$), and the orientation of the optical axis – θ^36^

$$R_{t}=\sqrt{p_{6}^{2}+p_{5}^{2}+p_{4}^{2}},\;\alpha_{t}=\frac{1}{3}|\alpha_{1}+\alpha_{2}+\alpha_{3}|,\;\theta =\frac{1}{2}\;\tan^{-1}[\frac{p_{5}}{p_{6}}].$$ (4)

In the above equation, the depolarization factors $\alpha_{1}$, $\alpha_{2}$, and $\alpha_{3}$ vary within the interval (−∞,0] for physically realizable differential Mueller matrices. The factor of 1/2 in the azimuth definition is related to a physical azimuth in the absence of a circular component. If one wants to relate to an orientation angle for the Poincaré sphere representation, then the factor of 1/2 should be omitted.

### 3.2 Lu–Chipman Decomposition

A decomposition algorithm, first proposed by Lu and Chipman^34^ and widespread in the polarimetric community, can also be applied for the analysis of the physically realizable Mueller matrices. It is based on a factorization of a sample’s Mueller matrix into the sequence of Mueller matrices of diattenuator $M_{D}$, retarder $M_{R}$, and depolarizer $M_{\Delta }$ (a.k.a forward decomposition^19^ – reversed decomposition also exists,^19^ but in this paper we only used the first one)

$$M=M_{\Delta }M_{R}M_{D}.$$ (5)

From the first row and column of M the net diattenuation D and net polarizance P can be obtained, as well as their vector forms^34^ ($m_{ij}$ denotes the elements of M)

$$D=\frac{1}{m_{11}}\sqrt{\underset{j}{\sum }m_{1j}^{2}}\equiv |\overset{\to }{D}|,\;P=\frac{1}{m_{11}}\sqrt{\underset{i}{\sum }m_{i1}^{2}}\equiv |\overset{\to }{P}|,\;i,j=2,3,4.$$ (6)

Once the diattenuation vector is obtained $M_{D}$ is given as^34^

MD=[1D→TD→mD],mD=1−D2I+(1−1−D2)D^D^T, (7)

where I is 3×3 identity matrix and D^ is the unit vector along $\overset{\to }{D}$. Knowledge of the diattenuation matrix facilitates the completion of this decomposition method by having^34^

$$MM_{D}^{-1}=M_{\Delta }M_{R}=[\begin{array}{cc} 1 & {\overset{\to }{0}}^{T}\\ \overset{\to }{P} & m_{\Delta }m_{R} \end{array}]=M^{\prime }.$$ (8)

The remaining part of the decomposition is based on solving the eigenvalue-eigenvector problem for the submatrix $m^{\prime }=m_{\Delta }m_{R}$, from which $m_{\Delta }$ and $M_{\Delta }$ can be reconstructed. Then, finally, $M_{R}$ is obtained by $M_{R}=M_{\Delta }^{-1}M^{\prime }$, while the net scalar retardance $R_{t}$, the net depolarization $\alpha_{t}$ and the orientation of the optical axis θ can be expressed as^34^

$$R_{t}=\cos^{-1}[\frac{tr(M_{R})}{2}-1],\;\alpha_{t}=1-\frac{\left|a|+|b|+|c\right|}{3},\;\theta =\frac{1}{2}\;\tan^{-1}[\frac{M_{R}(2,4)}{M_{R}(4,3)}],$$ (9)

where a, b, and c are the values of diagonal elements of the depolarizer matrix $m_{\Delta }$. Analogously, the physical azimuth is meaningful in the absence of circular retardance only.

## 4 Results and Discussion

**Fig. 4:**
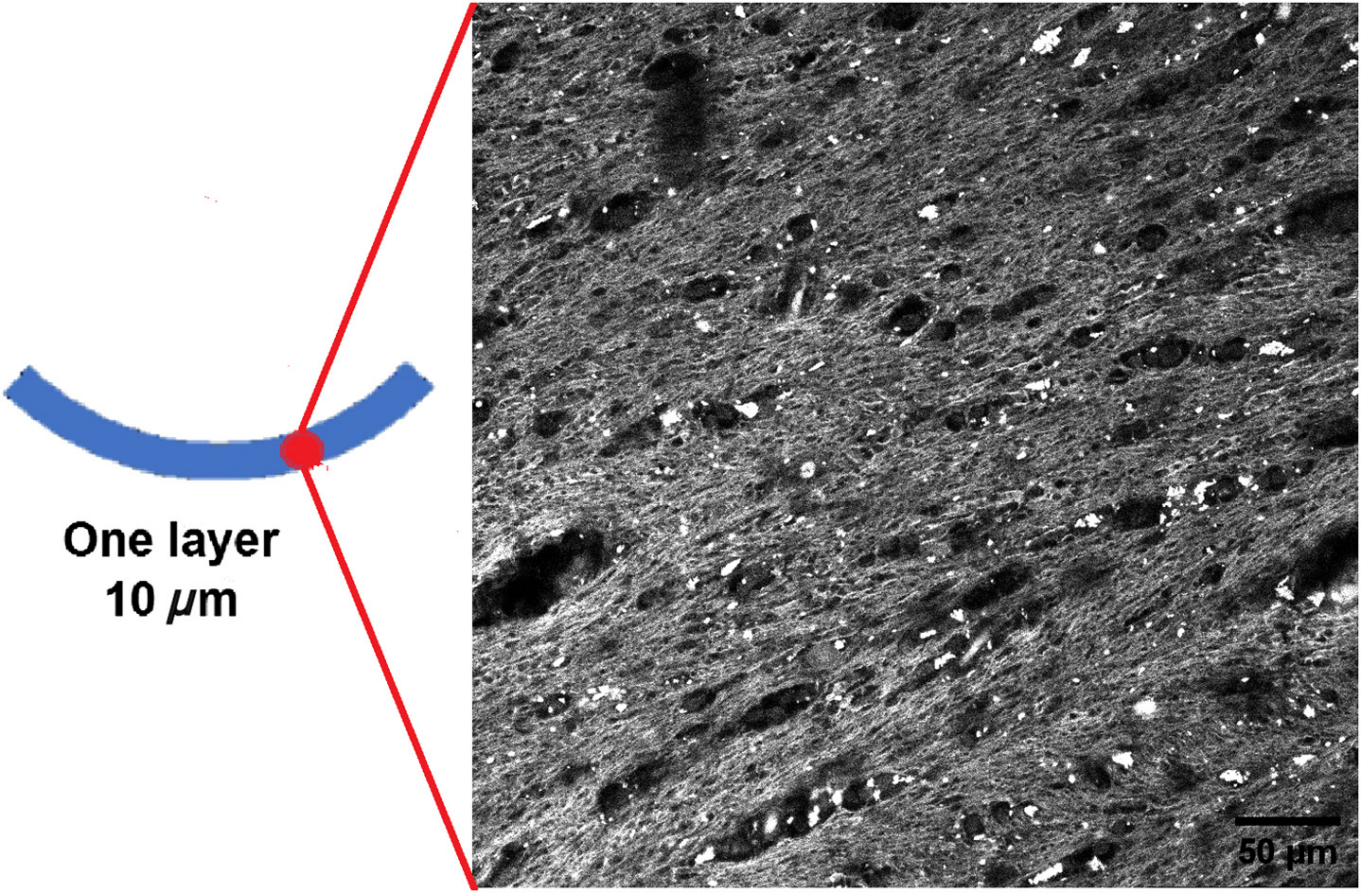
TPEF image of the corpus callosum section of 10  μm thickness, scale bar is 50  μm. The measurement location is shown by the red spot.

The corpus callosum in a human brain acts as a bridge connecting its two hemispheres. It consists of distinct fiber bundles that are oriented in a specific well-defined manner. In order to verify the presence and preferential orientation of the nerve fibers in studied samples, 10  μm thick corpus callosum section was first imaged with a custom-built upright multiphoton microscope, using 860 nm laser excitation and 25×, NA 1.05 objective lens with water immersion (XLPLN-MP, Olympus) as previously reported elsewhere.^42^ Two-photon excited fluorescence (TPEF) signal allowed the visualization of brain nerve fibers in thin brain sections without any staining by taking advantage of the slight fluorescence exhibited by fixed tissues. The histological sections were cut in a coronal plane and the fiber bundles have to be aligned along the “arc” (see Fig. 4, left). This preferential orientation of brain fibers is clearly seen in the TPEF image (Fig. 4, right) and it is consistent with the anatomy of corpus callosum.

It is worth to mention that the TPEF microscopy measurements were taken at a different site of the corpus callosum tissue compared to the Mueller microscopy measurements (see Fig. 2). However, Fig. 4 provides a ground truth evidence of the presence of nerve fibers that have a preferential alignment direction. Hence, apart from the depolarization properties related to light scattering, corpus callosum tissue should have an anisotropy of refractive index (so-called “form birefringence”).

### 4.1 Total Scalar Retardance

**Fig. 5:**
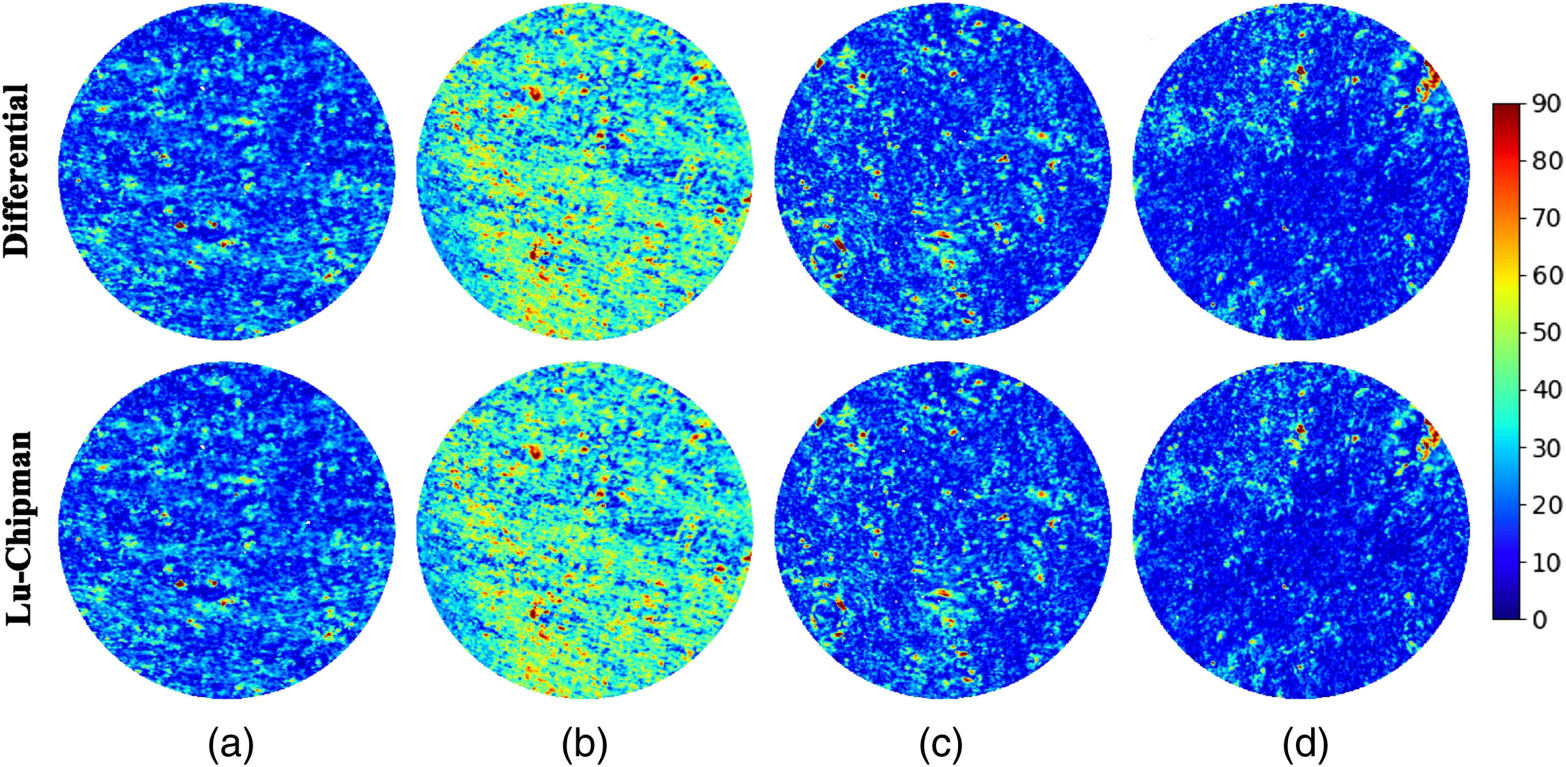
Net scalar retardance maps obtained with either differential (top row) or Lu–Chipman (bottom row) decomposition and corresponding to the different spatial arrangement of thin sections of corpus callosum: (a) one stripe (10  μm), two-layered stack (10  μm and 5  μm thick stripes) with (b) parallel overlap, (c) overlap at 45 deg, and (d) overlap at 90 deg.

We measured the Mueller matrix images of a single stripe of corpus callosum of 10  μm nominal thickness and calculated the maps of the retardance using either differential or Lu–Chipman decomposition applied pixel-wise. The corresponding maps are shown in Fig. 5(a).

**Fig. 6:**
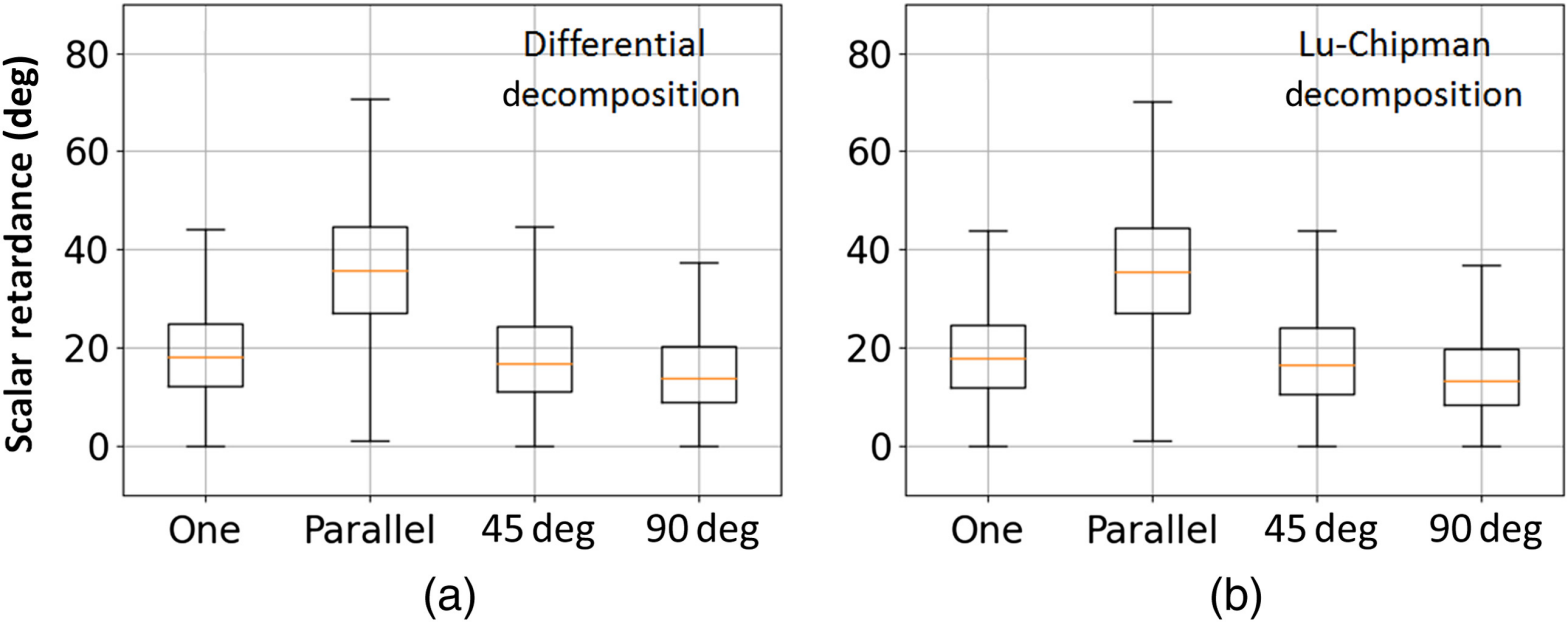
Box-whisker plots of the net scalar retardance obtained by (a) differential and (b) Lu–Chipman decomposition algorithms and corresponding to different spatial arrangement of corpus callosum stripes.

Then we measured the stack of two superimposed stripes of corpus callosum (10  μm and 5  μm of nominal thicknesses) that were arranged parallel, at 45 deg and 90 deg (see Fig. 2) and plotted the corresponding net scalar retardance maps as shown in Figs. 5(b)–5(d), respectively. The stack of two stripes of corpus callosum tissue of different thickness represents also an anisotropic medium and its polarimetric response varies with the relative orientation of two stripes. As can be seen in Fig. 5, both decomposition algorithms produce the same results. This is also confirmed by the statistical analysis of the corresponding images as illustrated by the box-whisker plots shown in Fig. 6. The addition of another corpus callosum stripe in parallel arrangement increases the optical path length of the transmitted detected signal compared to single stripe measurements and consequently increases the net scalar retardance. The median values of the corresponding distributions change from 18 deg to 35 deg [Figs. 6(a) and 6(b)]. Upon rotation of the top tissue stripe, the median value of scalar retardance decreases, reaching the lowest value for the mutually orthogonal configuration of two stripes. It is worth to mention that the latter configuration would result in complete phase retardance compensation, i.e., zero value of scalar retardance, for two identical corpus callosum tissue stripes of equal thickness. A preferential orientation of densely packed nerve fiber bundles within the imaging plane is detectable in Figs. 5(a) and 5(b), whereas it is almost completely lost in Fig. 5(d) for two superimposed stripes with mutually orthogonal orientation of the brain fibers causing the significant reduction of the net scalar retardance. Such results are consistent with the prior studies of the scattering anisotropic optical phantoms.^43^^,^^44^

### 4.2 Azimuth of the Optical Axis

**Fig. 7:**
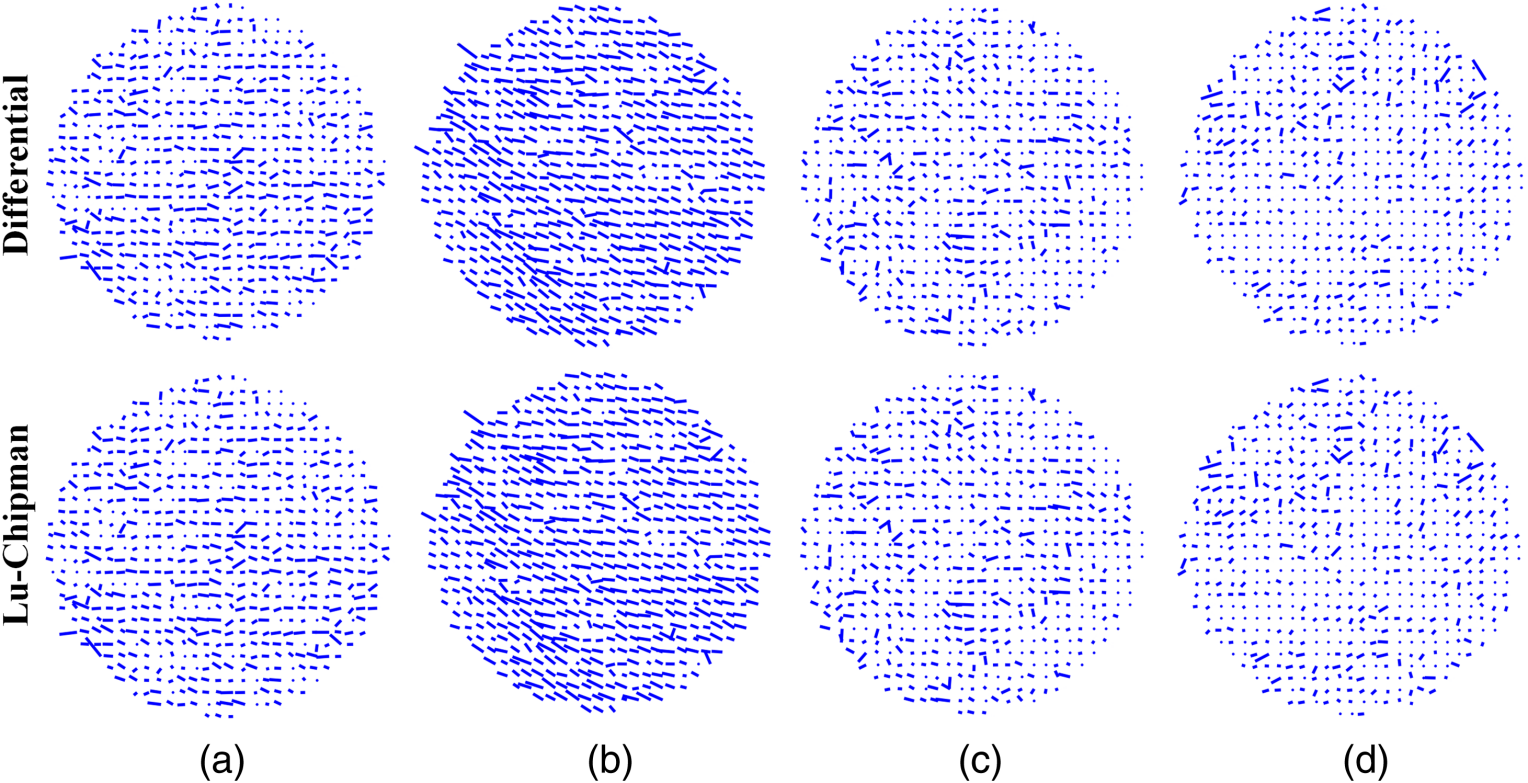
The quiver plots of the azimuth of the optical axis obtained with either differential (top row) or Lu–Chipman (bottom row) decomposition and corresponding to the different spatial arrangement of thin sections of corpus callosum: (a) one stripe (10  μm), two-layered stack (10  μm and 5  μm thick stripes) with (b) parallel overlap, (c) overlap at 45 deg, and (d) overlap at 90 deg. The sparse grid with the steps of 20 pixels was used for the visualization.

It was demonstrated that the myelinated nerve fiber bundles display negative form birefringence.^45^ The quiver plots in Fig. 7 show the orientation of the slow optical axis that is aligned with the orientation of corpus callosum fibers at each pixel of the image. The length of each stick is equal to the corresponding value of total scalar retardance R to the maximum value of R over all image pixels. As can be seen in Fig. 7 both decomposition algorithms produce almost identical results. Several zones in the image of the stack of two parallel superimposed stripes of corpus callosum demonstrate quite uniform azimuth distribution [see Fig. 7(b)], whereas the quiver plots of vector retardance in Figs. 7(a), 7(c), and 7(d) do not show any preferential orientation of the optical axis. This is explained by the presence of large number of pixels with the values of net scalar retardance below the measurement accuracy level of our instrument (∼2  deg−3  deg).^46^

**Fig. 8:**
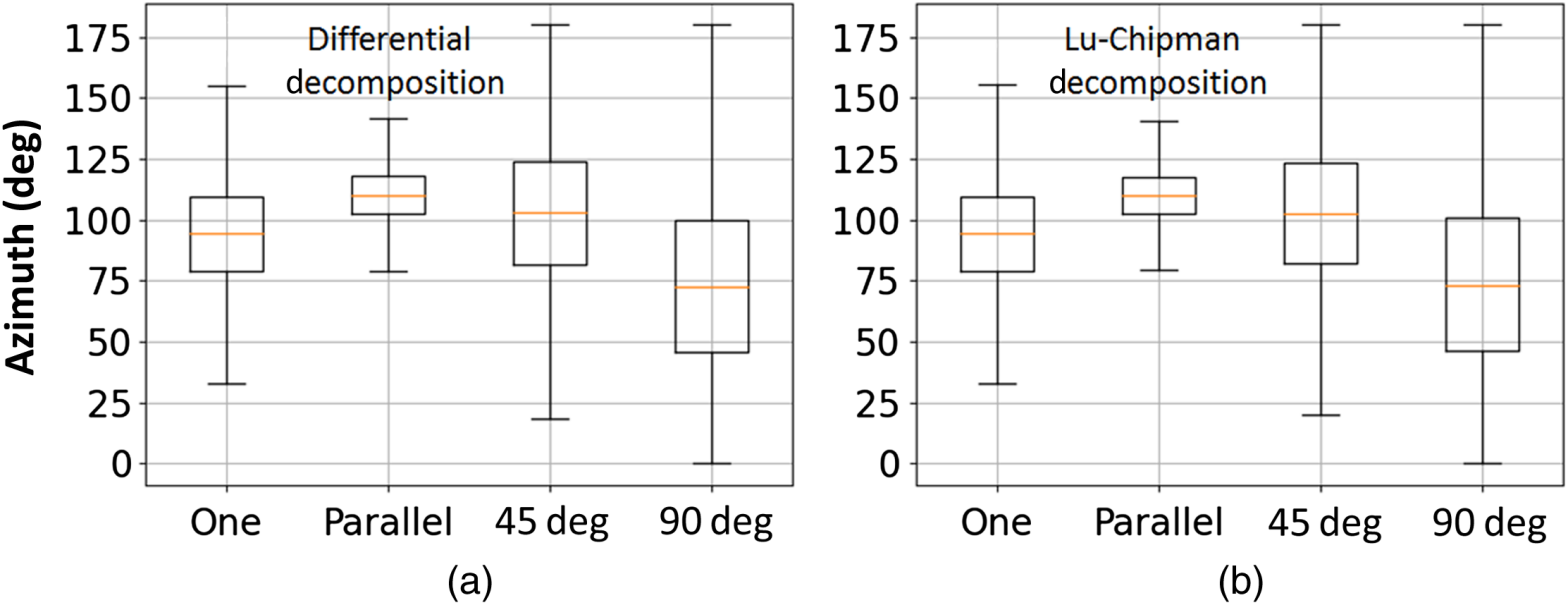
Box-whisker plots of the azimuth of the optical axis obtained by (a) differential and (b) Lu–Chipman decomposition algorithms and corresponding to different spatial arrangement of corpus callosum stripes.

The corresponding box-whisker plots of the azimuth of the optical axis are shown in Fig. 8. The median value of azimuth distribution for the single 10  μm thick stripe of corpus callosum is close to 90 deg, the dispersion of data is quite large. The latter is most probably related to the presence of holes in corpus callosum tissue (see Fig. 4). The azimuth value calculations in the “no tissue” zones are strongly affected by the measurement noise.

The box-whisker plots of the azimuth distribution for the stack of two parallel (10  μm+5  μm thick) stripes of corpus callosum demonstrate much smaller data spread with median value of ∼110  deg. The latter value is explained by the manual positioning of two layers stack on the sample holder. Parallel overlap of two corpus callosum stripes results in more narrow distribution of azimuth angle values because of reducing the number of “no tissue” zones in the measured sample and consequent increase of signal-to-noise ratio.

The spread of data is much larger for the distributions of the azimuth calculated for the corpus callosum stripes overlapped at 45 deg and 90 deg. The randomization of the azimuth values and the drop of total scalar retardance values serve as the indicators of the loss of optical anisotropy of the last two-layered stacks of corpus callosum.

### 4.3 Depolarization

**Fig. 9:**
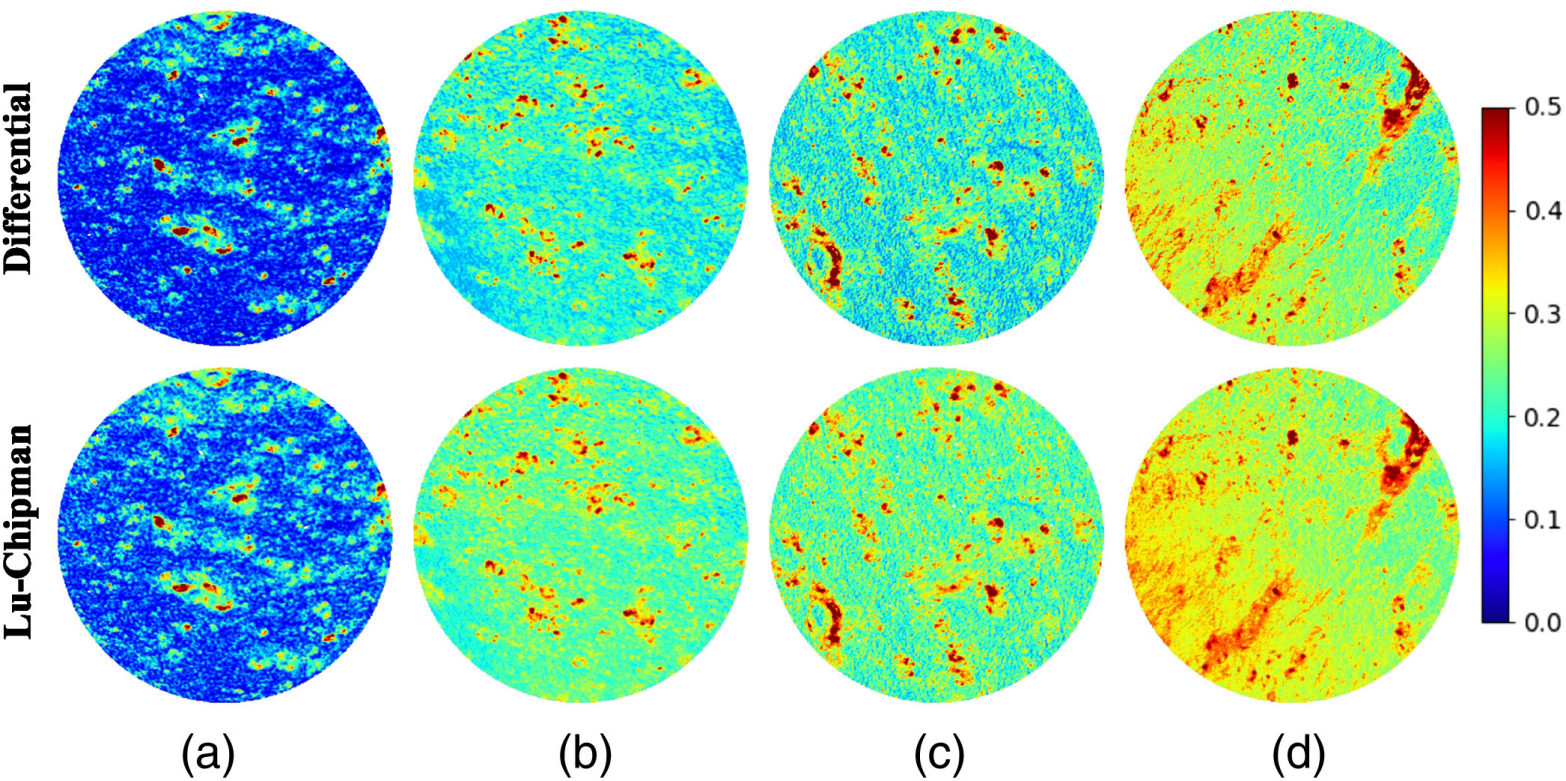
Total depolarization maps obtained with either differential (top row) or Lu–Chipman (bottom row) decomposition and corresponding to the different spatial arrangement of thin sections of corpus callosum: (a) one stripe (10  μm), two-layered stack (10  μm and 5  μm thick stripes) with (b) parallel overlap, (c) overlap at 45 deg, and (d) overlap at 90 deg.

The maps of the depolarization calculated using either differential or Lu–Chipman decomposition applied pixel-wise are shown in Fig. 9.

The lowest depolarization values account for a single layer of corpus callosum [see Fig. 9(a)], while the depolarization values increase for two superimposed stripes of corpus callosum regardless of their orientation [see Figs. 9(b)–9(d)]. An increase of physical thickness of measured sample related to the addition of a second tissue stripe leads to the increase in the number of scattering events and favor the loss of spatial coherence and hence, the depolarization of transmitted detected light. The above mentioned process does not depend on the orientation of fiber bundles, but depends only on their density and optical thickness of a sample. The rotation of the top slide with 5  μm thick stripe was performed manually. We attribute the slight increase in depolarization measured for the mutually orthogonal stripes [Fig. 9(d), left zone of the images] to the local variation of top tissue stripe thickness. This is supported by the drop in values of scalar retardance in the corresponding zones [see Fig. 5(d)].

**Fig. 10:**
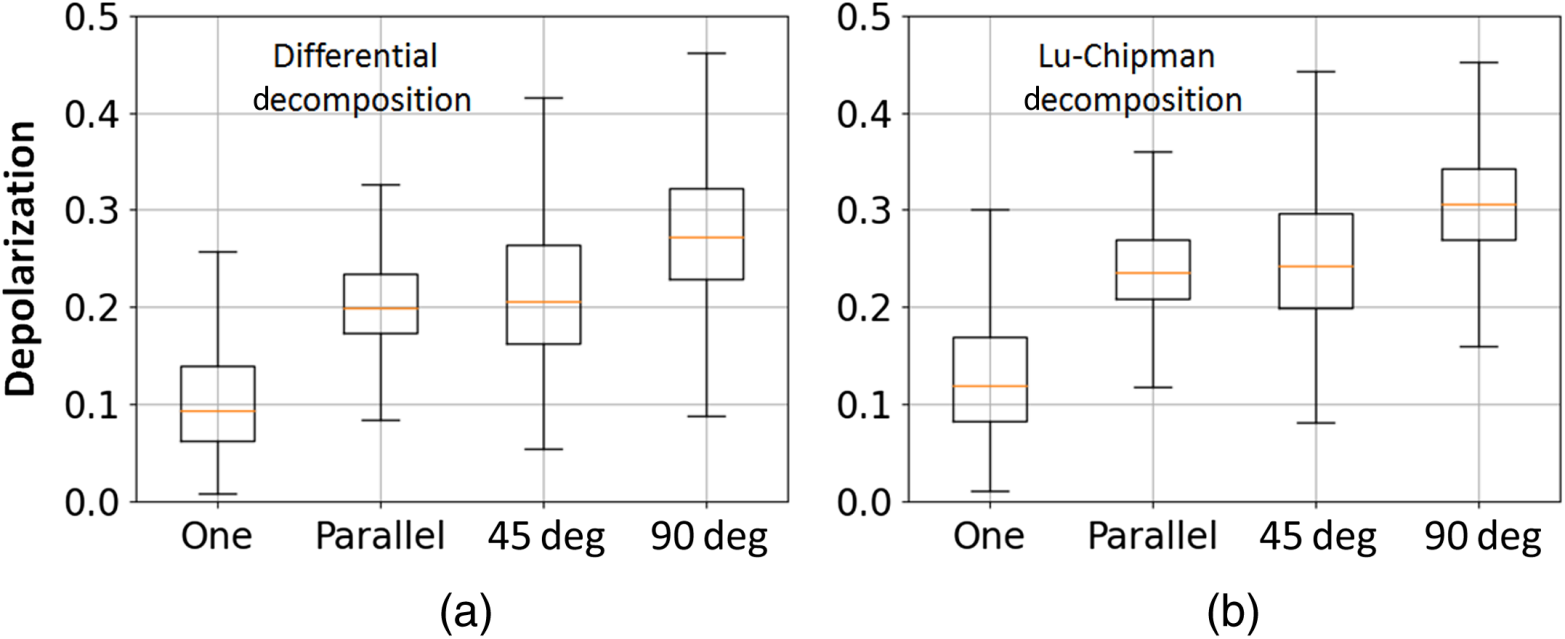
Box-whisker plots of the total depolarization obtained by (a) differential and (b) Lu–Chipman decomposition algorithms and corresponding to different spatial arrangement of corpus callosum stripes.

The box-whisker plots of the distributions of total depolarization are shown in Fig. 10. One can observe that slightly higher values of depolarization are obtained from the Lu–Chipman decomposition algorithm compared to the depolarization values obtained with the differential decomposition. It can be explained by the fact that $\alpha_{t}$ calculated with the Lu–Chipman decomposition varies between 0 for non-depolarizing sample and 1 for completely depolarizing sample, while the values of $\alpha_{t}$ calculated with the differential decomposition cover larger interval from 0 for non-depolarizing sample to +∞ for completely depolarizing sample.

## 5 Conclusions

In our study, the thin histological sections of human brain corpus callosum were arranged in different spatial configurations to measure their polarimetric response with the custom-built transmission Mueller microscope that was designed, aligned, and calibrated to measure the complete Mueller matrices of the samples under study. The two-layered stack (10  μm+5  μm) was thin enough to assure the collection of the substantial amount of transmitted light at the detector.

To extract the polarization and depolarization properties of the brain corpus callosum specimens, two different types of decomposition algorithms were used, namely the differential and Lu–Chipman decompositions. The former assumes the continuous variation of the polarization and depolarization properties of a medium, while the latter makes use of their sequential appearance. It makes the differential decomposition particularly suited for the post-processing of Mueller matrices of biological tissues that usually represent the complex structures with spatially intermixed polarimetric properties. However, the differential decomposition works well in transmission configuration, but may fail to process the Muller matrix data recorded in reflection, as for highly scattering media it may lead to the calculations of the logarithm of a negative number. The Lu–Chipman decomposition works well on the Muller matrix data recorded in both transmission and reflection configurations. The latter configuration is the only one relevant for the clinical applications of the imaging Mueller polarimetry operating in a visible wavelength range. Hence, the equivalence of the results obtained with the Lu–Chipman and differential decompositions in transmission configuration supports the use of the former algorithm for the interpretation of the Mueller matrix data of biological tissues recorded in reflection.

It was found out that the net scalar retardance of the stack of two thin stripes of corpus callosum decreases with the increase of fiber crossing angle between two overlapped sections of brain corpus callosum tissue. This effect is explained by the partial compensation of the phase shift acquired by polarized light passing through the first stripe by the phase shift of the opposite sign acquired through the second stripe with different preferential orientation of the fibers

Our prior studies demonstrated that the maps of the azimuth of the optical axis allow us monitoring a preferential 2D orientation of the fiber bundles at the site of the measurement in reflection configuration. The analysis of the azimuth images of 15  μm thick two-layered stack of corpus callosum stripes measured in transmission shows that the optical anisotropy of such sample is almost completely erased, when the preferential directions of brain fibers within each corpus callosum stripe are not parallel to each other. The randomization of azimuth values accompanied by the drop of corresponding values of scalar linear retardance is also attributed to the presence of crossing nerve fibers. Thus, the presence of crossing nerve fibers within the depth of few microns (at least) may be detected in polarimetric maps of brain white matter measured in reflection configuration.

Two-layered stack of corpus callosum stripes (10  μm+5  μm thickness) significantly depolarizes transmitted light at different spatial arrangement of the stripes. The total depolarization was found to be almost insensitive to the brain fibers orientations, but dependent on sample’s thickness. Further studies are needed to check whether the depolarization signature from brain tumor tissue is different from that of zones of crossing fibers in healthy brain tissue.

Similar values of scalar retardance and azimuth of the optical axis were obtained for all spatial arrangement of corpus callosum sections with both decomposition algorithms, while higher values for the total depolarization parameter were obtained with Lu–Chipman decomposition. Hence, the choice of decomposition algorithm seems to be of non-negligible importance for the final results and the decomposition algorithm should be selected properly, in accordance to the experimental configuration, samples’ type and initial assumptions.

The results of our studies demonstrate that significant drop of retardance values and randomization of the azimuth in combination with almost constant depolarization values (variation <15%) were observed in the corresponding images of healthy brain white matter with crossing nerve fibers. Hence, further studies of depolarization from brain tumor zone are needed as the image of this parameter in combination with the images of scalar linear retardance and the azimuth of the optical axis may become a decisive observable to differentiate the tumor zone from the zone of healthy brain white matter with underlying crossing nerve fibers that are located below the imaged surface of brain but within the probed volume of brain.

Achieving this goal will be of significant importance for *in vivo* applications of imaging Mueller polarimetry in neurosurgery. The estimation of light penetration depth within the white matter of brain in reflection configurations is the subject of our ongoing studies.

## Data Availability

Data underlying the results presented in this paper are not publicly available at this time but may be obtained from the authors upon reasonable request.
